# 

**Published:** 1911-02-11

**Authors:** 


### THE HOSPITAL

Telegraphic Address: Ospedale, London.

THE MODERN MEDICAL AND INSTITUTIONAL JOURNAL.

Telephone Number: 2734 Gerrard.

NEW SERIES. No. 210, Vol. VIII.

No. 1282, Vol. XLIX.

SATURDAY, FEB. 11TH, 1911.

PRICE THREEPENCE

